# Laparoscopic pancreaticodoudenectomy: An excellent approach in elderly patients, a multicenter, comparative study

**DOI:** 10.1097/MD.0000000000022175

**Published:** 2020-09-18

**Authors:** Maher Hendi, Yiping Mou, Chao Lu, Yu Pan, Bin Zhang, Ke Chen, Xiaowu Xu, Renchao Zhang, Yucheng Zhou, Weiwei Jin

**Affiliations:** a Zhejiang University, School of Medicine; b Department of Gastrointestinal and Pancreas Surgery, Zhejiang Provincial People's Hospital; c Department of General Surgery, Sir Run Run Shaw Hospital, School of Medicine, Zhejiang University, Hangzhou, China.

**Keywords:** comparative study, laparoscopic pancreaticoduodenectomy, short-term outcomes’, surgical operation, safety, elderly

## Abstract

**Background:**

Management of malignant diseases in elderly patients has become a global clinical issue because of increased life expectancy worldwide. Advancements in surgical techniques and perioperative management have reduced age-related contraindications for LPD. Past articles have reported that elderly patients undergoing laproscopic pancreatoduodenectomy (LPD) are at an increased risk compared to younger patients. The aim of this article is to compare a multicenter center risk of LPD in elderly and nonelderly patients.

**Methods:**

Retrospective review (n = 237) of perisurgical outcomes in patients undergoing LPD during the months of September 2012 to December 2017. Outcomes in elderly patients (aged ≥75 years) were compared with those in nonelderly patients.

**Results:**

Transfer to ICU was more frequent in elderly patients (odds ratio [OR] 6.49, *P* = .001) and the mean hospital stay was longer (21.4 days compared with 16.6 days), (*P* *=* .0033) than for nonelderly patients. There was no statistically significant difference in operation time (*P* *=* .494), estimated blood loss (*P* *=* .0519), blood transfusion (*P* *=* .863), decreased gastric emptying (*P* = .397), abdominal pain (*P* *=* .454), food intake (*P* = .241), time to self-ambulation (*P* *=* 1), reoperation (*P* *=* .543), postoperative pancreatic fistula (POPF) grade A (*P* *=* .454), POPF grade B (*P* *=* .736), POPF grade C (*P* *=* .164), hemorrhage (*P* *=* .319), bile leakage (*P* *=* .428), infection (*P* *=* .259), GI bleeding (*P* *=* .286), morbidity (*P* *=* .272) or mortality (*P* *=* .449) between the 2 groups.

**Conclusions:**

Elderly patients who underwent LPD in this study had good overall outcomes after LPD that were similar to young patients. The perioperative and long-term outcomes of LPD are not worse. Rates of ICU admission and hospital stays increased in elderly patients undergoing LPD when compared with nonelderly ones. LPD can be performed on elderly patients with similar outcomes as younger patients; therefore, age itself should not be a contraindication for LPD for pancreatic cancer, but it suggests that elderly patients with comorbidities should be more stringently selected for surgery.

## 1 Introduction

Management of malignant diseases in elderly patients has become a global clinical issue because of the increased life expectancy in many countries.^[1,2]^ Laparoscopic pancreatoduodenectomy (LPD) was first introduced in 1994^[3]^ in recent years; this approach has been shown to be safe and feasible when performed by experienced surgeons in centers with high volumes of cases.^[4]^ Laparoscopic surgery is now a widely utilized technique for the treatment of a variety of both benign and malignant diseases because it is associated with a lower degree of invasion, less pain, and shortened postoperative hospital stay than open surgery. Laparoscopic surgery bestows several advantages when compared to open surgery in elderly patients undergoing pancreatoduodenectomy (PD).^[5–8]^ In addition, the 2 have similar oncological results.^[5,8,9]^ Laparoscopic surgery has, in a number of studies, been shown to result in less postoperative pain, fewer wound complications, shorter hospital stays, decreased pancreatic fistula rates, and decreased surgical morbidity and mortality.^[5,10–12]^ However, those selected for laparoscopic PD are predominantly otherwise healthy patients under the age of 75; thus, the extrapolation of results from such trials to daily practice necessitates careful consideration. Longer operation times and higher incidences of organ injury^[13–16]^ are of particular concern when considering LPD surgery for elderly patients.^[17]^ One retrospective analysis of robot-assisted PDs concluded that the procedure can be performed safely in elderly patients with mortality, morbidity, and outcomes comparable to those in younger patients.^[18]^ Sperti et al showed that outcomes after pancreatectomy were not markedly different in octogenarians than in younger patients^[19]^ and Maehara et al found no statistically significant difference in the mortality rate or overall morbidity rate in patients undergoing PD for periampullary tumors above and below the age of 75.^[20]^

In all, 237 patients underwent LPD in a relatively short period, 5 years to be exact. Patients aged 75 years and older comprised nearly 26% of the analysis, which is comparable to the age distribution of the world's population and the increasing age at which pancreatic cancer is now being diagnosed. Following this, a retrospective review (n = 237) of perisurgical outcomes for elderly (aged ≥75 years) and nonelderly LPD patients was carried out.

In our multicenter favors, a laparoscopic approach for pancreatic cancer resection, regardless of patient age. However, a huge body mass is a contraindication for the procedure. Consequently, approximately 80% of our pancreatic cancer patients underwent laparoscopic surgery, minimizing surgical approach selection bias in this study

This study aims to evaluate the safety of LPD for elderly patients with pancreatic disease by retrospectively analyzing the medical records of elderly patients aged ≥75 years who underwent surgery between September 2012 and December 2017 in our center.

## 2 Materials and methods

### 2.1 Patients Selection

We analyzed data from 237 consecutive patients who underwent LPD in 2 centers between September 2012 and December 2017. The centers were Zhejiang University Sir Run Run Shaw Hospital and Zhejiang Provincial People's Hospital. Sixty-one of the patients were elderly (aged ≥75 years). The collected data were retrieved from prospectively maintained databases and included baseline patient characteristics. Results of pathological examinations were used as an indicator of preoperative factors, based on the assumption that preoperative findings would correlate with postoperative staging. The Institutional Review committee of Zhejiang Provincial People's Hospital and Zhejiang University approved this study.

The patients were followed up at their respective institution. Most of the patients were traditionally observed according to a protocol similar to the Chinese guidelines,^[21,22]^ including the commonly used ultrasonography, novel imaging methods including magnetic resonance imaging (13.9%), positron emission tomography/computed tomography (1.8%), and EUS (5.6%) were not widely used in our population. Only 39.7% of cases were histologically verified (surgery with histologic diagnosis 31.0%, cytological diagnosis 8.7%, surgery without histologic diagnosis 12.1%, and clinical diagnosis 48.2%). Overall, 30.0% of patients underwent curative-intent operation, and only 9.8% of patients received comprehensive treatment.

### 2.2 Surgical procedure

One experienced surgeon performed most of the operations, Professor Yiping Mou**.** The rest were carried out by surgeons with sufficient laparoscopic pancreatic surgery experience under supervision. Our surgical procedures called (Wu Kong Zi) have been previously described.^[23]^ The procedures for patients with resectable PD were performed using general anesthesia with the patient in the supine position and legs apart. “Five Trocars,”^[23]^ were used for the procedure. The trocars were placed as follows; 1 initial 10 mm trocar was placed below the umbilicus for laparoscopy. The other 4 trocars, one 12 mm in diameter and 3 5 mm in diameter, were inserted into the left upper flank, left flank, right upper flank, and right flank quadrants, respectively. The 5 trocars were arranged in a V formation. However, for patients who had SMV encasement which made creating the retropancreatic tunnel difficult (borderline resectable pancreatic cancer), we used the “Easy First” strategy to perform LPD.^[24]^ The definition of mesopancreas used was: the soft connective tissue along the celiac axis, superior mesenteric vessels and the uncinate process of the pancreas, especially the lymphatic and nervous structures of the retroperitoneal margin, as has been previously reported.^[25]^ After the specimens were removed from the enlarged umbilical port, a frozen section was sent off to confirm the negative margins. Child's reconstruction was then performed in a complete laparoscopic manner following individual construction. Laparoscopic pancreaticojejunostomy (LPJ) was performed using the duct-to-mucosa method. If the diameter of MPD was between 2 and 5 mm, LPJ was carried out using interrupted sutures of 4 to 6 stitches with stents of the proper diameter. As for MPDs >5 mm, running sutures were used without stents, and nonabsorbable sutures were used instead of absorbable sutures. Laparoscopic choledochojejunostomy was performed with running sutures if the CBD was >8 mm or with interrupted sutures if CBD was <8 mm. As for the laparoscopic gastrojejunostomy, we used an endoscopic linear stapler to perform a side-to-side anastomosis with running sutures to close the opening.

### 2.3 Statistical analysis

A comparison of each variable at baseline and post treatment was carried out to identify statistically significant differences between elderly and nonelderly patients. Continuous data were expressed as mean (SD) or mean (SEM) or median (interquartile range, IQR) and the means were compared using 2 independent samples of Student *t* test. Categorical data were compared using the *χ*^2^ test or Fisher exact probability test. The Mann-Whitney *U* test was used for abnormally distributed variables. Statistical significance was defined at the level of 0.05. All analyses were performed using statistical software SPSS 19.0 (SPSS Inc, Chicago, IL).

## 3 Results

### 3.1 Patients

A total of 237 patients underwent LPD in our centers between 2012 and 2017, of which 61 (25.74%) were elderly. All procedures were performed in a purely minimally invasive fashion. All operations were initiated laparoscopically. There were 144 male and 93 female patients with a median age of 73 (range 19–92) years and a median body mass index of 22.65 kg/m^2^. However, comorbidities were more common in elderly (68.9%) than in nonelderly patients (34.6%) (odds ratio [OR] 4.17, *P* = .0001). Basic demographics for the entire cohort are summarized in Table 1.

**Table 1:**
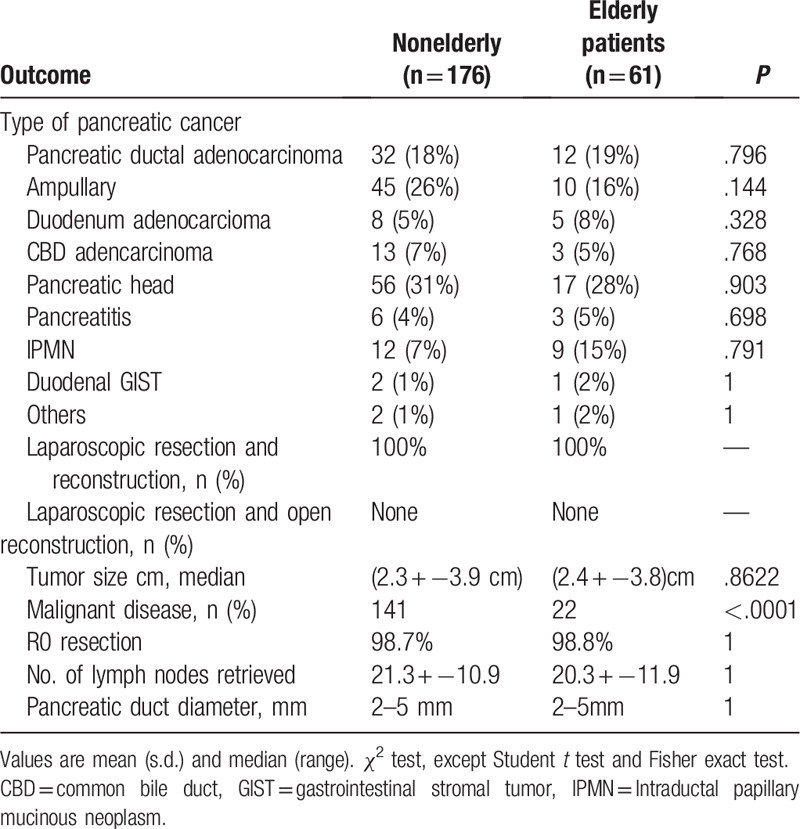
Histopathology data.

Table 2: Baseline characteristics of entire patients cohort

**Table 2:**
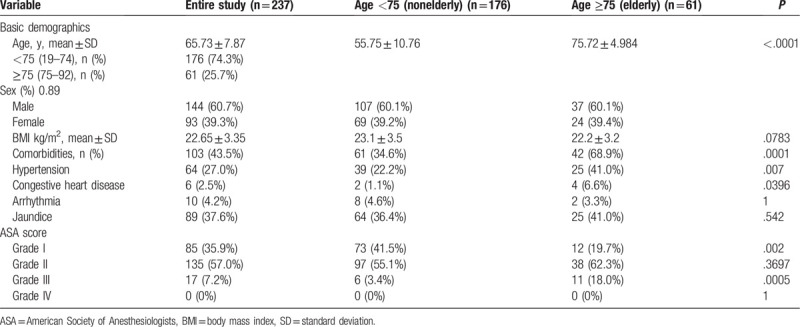
Baseline characteristics of entire patients cohort.

### 3.2 Histopathological analysis

Operative details are shown in Table 1. Tumors sized >5 cm and 173 (98.7%) versus 60 (98.8%) underwent an R0 resection so the rate of R0 resection was similar in the 2 groups (Table 1). Most of the patients (n = 141 [80%] vs 22 [36%]) were diagnosed with malignant disease. The mean number of lymph nodes removed was similar in the 2 groups; lymph nodes retrieved in the LPD nonelderly group and LPD elderly groups were (21.3 **±** 10.9 vs 20.3 **±** 11.9), respectively (*P* = 1). Median pancreatic duct diameter was >5 mm (*P* = 1). The histopathological outcomes can be seen in Table 1.

#### 3.2.1 Operative Outcomes

Operative details are shown in Tables 1 and 3 and Table 4. The definition of mortality used in this article was either death before being discharged from the hospital or within 30 days of surgery. Surgical morbidity was also measured for 30 days after the operation and was based on hospital records, readmissions, and routine follow-up information. Wound infection was defined by any superficial, deep, or organ infection with or without an associated wound and/or fascial dehiscence. Complications were graded on severity according to the Clavien–Dindo classification. Major morbidity was defined as a complication of Clavien–Dindo Grade IIIb or higher. Decreased gastric emptying (DGE), post-pancreatectomy hemorrhage, and POPF were classified based on the International Study Group of Pancreatic Surgery definitions.^[26–28]^ Reoperations and readmissions were defined as those occurring within 30 days of surgery. Reoperations and re-admissions outside the study institution were confirmed through outside institutional charts that were also reviewed to capture additional morbidity information related to these occurrences. Records of any unplanned admissions to the ICU of either the index hospital admission or readmission were also obtained. Postoperative blood transfusion was defined as any receipt of packed red blood cells (PRBC) during the course of hospitalization. Length of stay was the duration of hospitalization from the date of surgery until the time of discharge. There were no statistically significant differences in operation time (*P* *=* .494), estimated blood loss (*P* *=* .0519), or blood transfusion (*P* *=* .863). Intraoperative outcomes can be seen in Table 3.

**Table 3:**
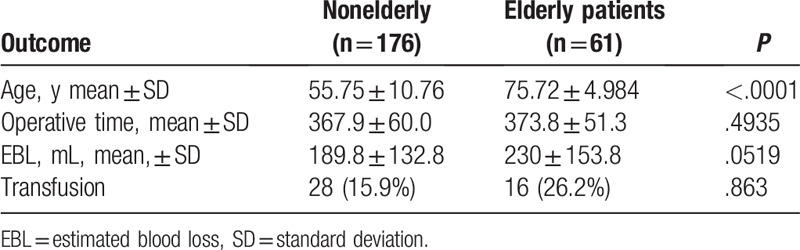
Intraoperative Outcomes for elderly versus nonelderly patients.

**Table 4:**
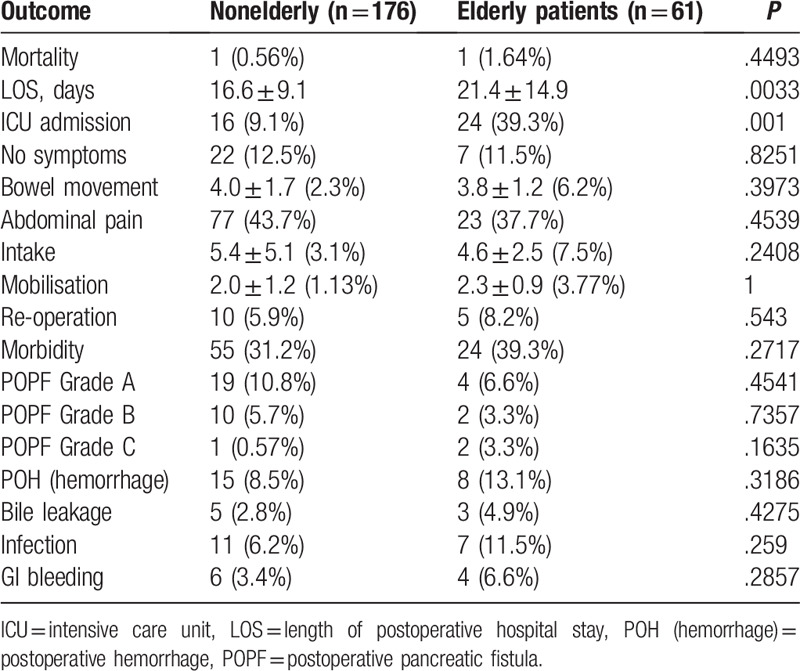
Postoperative outcomes for elderly patients versus non-elderly patients.

The in-hospital mortality rate was 0.84% (n = 2). Postoperative complications occurred in 79 patients (33%). Urgent reoperation was required in 15 patients (6%). Bile leakage occurred in 8 patients the rate was 3% (n = 8). In elderly patients, transfers to the ICU were more frequent (odds ratio [OR] 6.49, *P* *=* .001) and mean hospital stay was prolonged (21.4 days compared with 16.6 days, [*P* *=* .0033]) when compared with that of nonelderly patients. There was no statistically significant difference in DGE (*P* *=* .397), abdominal pain (*P* *=* .454), food intake (*P* *=* .241), time to self-ambulation (P = 1), reoperation (*P* *=* .543), POPF grade A (*P* *=* .454*)*, POPF grade B (*P* *=* .736), POPF grade C (*P* *=* .164), hemorrhage (*P* *=* .319), bile leakage (*P* *=* .428), infection (*P* *=* .259), gastrointestinal bleeding (*P* *=* .286), morbidity (*P* *=* .272), and mortality (*P* *=* .449) between the 2 groups. Postoperative outcomes can be seen in Table 4.

## 4 Discussion

In an increasingly aging society, laparoscopic surgery is being employed more frequently on elderly patients. However, only a limited number of studies have been conducted on laparoscopic surgery in elderly patients with pancreatic cancer. Compared to younger patients, elderly patients are more likely to have existing conditions when undergoing surgery, as well as reduced major organ function and reduced functional reserves for invasive surgery. This study showed that other than an increase in ICU transfer rate (OR 6.49, *P* = .001) and an extended length of hospital stay (21.4 days vs 16.6 days; *P* = .0033, elderly vs nonelderly), there were no significant statistical differences in any of the other outcomes, including morbidity and mortality, between elderly and nonelderly patients. These results are supported by a number of studies in relation to both postoperative morbidity and mortality.^[5,12,20]^ However, other articles have reported an increase in postoperative mortality and complications in elderly patients.^[7,18,29]^ A recent meta-analysis of >5000 patients demonstrated that patients 76 to 80 years’ old undergoing PD had increased postoperative mortality rates compared to younger patients.^[29]^ In addition elderly patients (when defined as aged >80 years) were found to have an increased risk for postoperative complications when compared with nonelderly patients.^[29]^ Elderly patients (aged >75 years) were also found to have increased risk for pulmonary complications compared with nonelderly patients.^[29]^ One possible reason for this disparity could be the definition of elderly patients used in the aforementioned articles, which was older. However, another retrospective review, which defined elderly as >70 years, found that elderly pancreatoduodenectomy patients (n = 860) were more likely to experience postoperative cardiorespiratory complications.^[5]^ This difference could be due to our study cohort being of an insufficient size to reveal statistically significant differences for such rare events. For example, total mortality in our study was just 0.84%. It is possible that this very low mortality rate may reflect an increase in skill of laproscopic surgeons in recent years. Our study demonstrated an increased risk of ICU admission and an increased length of hospital stay for elderly patients. However, this could be due to the fact that admission to ICUs and hospital discharge decisions are made partly dependent on a patient's age, which would make it a confounding factor. Our study supported Buchs et al's comparison of elderly patients (defined as those aged >70 years) and nonelderly patients undergoing robotic PD in that it identified no statistically significant differences in operative time, blood loss, postoperative mortality, or overall morbidity between the 2 groups.^[18]^ Limitations of our study and previous ones executed in a similar manner are found in their retrospective nature, which makes them susceptible to selection bias.

## 5 Conclusion

In our conclusion, we report our experience over 5 years with 237 laparoscopic resection cases including 61 cases with patients older than 75 years. Laparoscopic pancreatoduodenectomy is extremely challenging to perform. Our article suggests that neither morbidity nor mortality increased in elderly LPD patients when compared to nonelderly patients. Elderly patients experienced a higher admittance rate to the ICU and a longer hospital stay post operation; LPD is associated with lower overall complications, especially infections and bleeding. No statistically significant differences were found in any other complications assessed in this study. Therefore, age itself should not be a contraindication to LPD for pancreatic cancer, but it suggests that elderly patients with comorbidities should be more stringently selected for surgery.

## Author contributions

MH, YPM wrote the manuscript, YPM, XWX, RCZ, MH and YCZ performed the operations, CL, CK, MH,YP and BZ reviewed the medical records and collected data. All authors read and approved the final manuscript.

## Abbreviations

Abbreviations: BMI = body mass index, GI = Gastric-intestinal, LPD = laparoscopic pancreaticoduodenectomy, PD = pancreaticoduodenectomy, POPF = postoperative pancreatic fifistula.
